# A Pipeline to Call Multilevel Expression Changes between Cancer and Normal Tissues and Its Applications in Repurposing Drugs Effective for Gastric Cancer

**DOI:** 10.1155/2020/3451610

**Published:** 2020-08-05

**Authors:** Wei Gao, Jianwei Yang, Changhua Zhuo, Sha Huang, Jinyuan Lin, Guangfeng Wu, Min Zhou

**Affiliations:** ^1^Departments of Internal Medicine-Oncology, Fujian Cancer Hospital & Fujian Medical University Cancer Hospital, Fuzhou, Fujian 350014, China; ^2^Departments of Gastrointestinal Surgical Oncology, Fujian Cancer Hospital & Fujian Medical University Cancer Hospital, Fuzhou, Fujian 350014, China

## Abstract

Differential gene analyses on gastric cancer usually focus on expression change of single genes between tumor and adjacent normal tissues. However, besides changes on single genes, there are also coexpression and expression network module changes during the development of gastric cancer. In this study, we proposed a pipeline to investigate various levels of changes between gastric cancer and adjacent normal tissues, which were used to repurpose potential drugs for treating gastric cancer. Specifically, we performed a series of analyses on 242 gastric cancer samples (33-normal, 209-cancer) downloaded from the cancer genome atlas (TCGA), including data quality control, differential gene analysis, gene coexpression network analysis, module function enrichment analysis, differential coexpression analysis, differential pathway analysis, and screening of potential therapeutic drugs. In the end, we discovered some genes and pathways that are significantly different between cancer and adjacent normal tissues (such as the interleukin-4 and interleukin-13 signaling pathway) and screened perturbed genes by 2703 drugs that have a high overlap with the identified differentially expressed genes. Our pipeline might be useful for understanding cancer pathogenesis as well as gastric cancer treatment.

## 1. Introduction

Despite the development of science and technology, cancer is still a major disease we have to face. According to statistics, each year, more than 18 million new cancer cases are diagnosed worldwide. Among the living patients who have been diagnosed with cancer, about 44 million patients are told that they have less than five years of life. Cancer is also a disease with a high mortality rate. Nearly 10 million people die from cancer each year [1]. Among various cancers, gastric cancer is one of the most prevalent ones and gastric cancer patients mostly suffer from the poor prognosis of malignancy. Though there are a few anticancer drugs specifically designed for gastric cancer, more novel drugs are required to treat gastric cancer patients with different disease status.

Through the screening and testing of model organisms, more and more potential anticancer drugs have been discovered. Since most potential anticancer drugs are identified in model organisms with little human data support, their effectiveness in promoting human health remains unknown, and this uncertainty brings costly clinical trials to the pharmaceutical industry [2].

In the past 60 years, only about 130-180 kinds of anticancer drugs have been approved by the US FDA. There are about 1,300 to 1,500 kinds of various anticancer drug preparations formulated with these drugs. The research and development of new drugs and rational and effective medication guidance are still a big scientific problem.

At present, the most common drug development of gastric cancer is mainly through a large number of experimental screening, and its strategy may be slightly different, such as using some of the histological data for correlation, so as to achieve better efficacy [3]. But this method is very expensive for early drug screening, especially when the number of potential drugs is large. It has also been reported that using drug data and clinical case characteristics for machine learning to build software to guide drug use [4], such strategies have limited understanding of the pathogenesis of cancer, and the precise treatment of cancer is limited.

Several methods of computer-aided anticancer drug development have been reported. For example, many proteins have been resolved by X-ray or nuclear magnetic resonance (NMR) spectra and are available from the Open Access Protein Database (http://www.rcsb.org). This information enables researchers to understand and characterize many physiological processes based on the interactions between proteins or between proteins and small molecules (ligands), such as when drugs bind to targets. In addition to the 3D structure of the molecule, van der Waals radii, the parameters of covalent bonds, torsions, and dihedral angles were also considered, so people can quickly develop some anticancer drugs based on specific receptor proteins on the surface of cancer cells [5].

For the effective use of anticancer drugs, a more elegant solution does not seem to have been proposed at present. In this article, we will provide a new idea for cancer research and drug treatment. Here, we will compare gene expression before and after cancer and changes in gene regulatory networks, construct key sets of cancer genes, and provide potential medication guidance through drug regulatory data. In this article, we will focus on gastric cancer.

## 2. Materials and Methods

### 2.1. Data Collection and Processing

#### 2.1.1. Gene Expression Data

The sample data (gastric cancer) are all from The Cancer Genome Atlas (TCGA) database. We used the R package of “*RTCGAToolbox*” to download the RNA sequencing data (reads counts data) of gastric cancer. After excluding some irregular sample data, we obtained a total of 242 gastric cancer samples (33 normal samples, 209 cancer samples).

#### 2.1.2. Drug Regulatory Gene Data

Justin Lamb once proposed a network pharmacogenomic approach based on the concept of Connectivity Map (CMap) [6]. The CMap project contains more than 6,000 drug-perturbed gene expression profiles generated from multiple human cell lines, including more than 1,309 compounds. CMap can be used to query gene expression profiles related to various diseases, thereby identifying drugs that may “reverse” the expression of these genes. Such drugs have potential application value for treating corresponding diseases. The CMap concept has been successfully applied to disease research [7, 8], and these successes have stimulated researchers to build a larger-scale perturbation-induced expression database, e.g., The Library of Network-Based Cellular Signatures (LINCS) Program [9], and a crowd extracted expression of differential signatures (CREEDS) [10]. Here, we used 8590 drug perturbation-induced gene expression signatures collected in Crowd Extracted Expression of Differential Signatures (CREEDS) for our analysis (http://amp.pharm.mssm.edu/creeds).

### 2.2. Analysis of Gene Expression Differences between Cancer and Normal Samples

Read counts were used to call differential expression genes by DESeq2 [11] between cancer and normal samples (adjusted *p* value less than 0.05 was set as threshold). Before using DESeq2 for analysis, we used principal component analysis (PCA) to screen all samples of gastric cancer, excluding some outlier points to reduce sample disturbance.

### 2.3. Construction of Cancer and Normal Gene Coexpression Network

We divided gastric cancer samples into normal and cancer samples, and we removed the abnormal samples to build a coexpression network through hierarchical clustering provided by weighted gene correlation network analysis (WGCNA) [12]. The soft threshold is set as follows: gastric cancer (normal-6, cancer-6).

### 2.4. Analysis of Gene Regulatory Networks

We can obtain the clustered gene modules through weighted gene correlation network analysis (WGCNA). The correlation of gene expression within the modules is relatively high, which may belong to the same regulatory subnetwork. We select the modules clustered by the normal sample group in gastric cancers for functional enrichment analysis, find cancer-related functional pathways, and use the genes in the module as a benchmark to compare the changes of these genes in the corresponding cancer sample groups to explore cancer gene regulation changes from normal samples.

### 2.5. Analysis of Potential Applicability Drugs

8590 drug perturbation-induced gene expression signatures collected in Crowd Extracted Expression of Differential Signatures (CREEDS) were used in our analysis. For signatures from CREEDS, we use Fisher's exact test to rank them, and we calculated the significance of the overlap between up- and down-regulated genes in normal and cancer samples with drug perturbation-induced up- and down-regulated genes. A drug was ranked to the top if drug-induced genes significantly overlapped with differentially expressed genes in normal and cancer samples.

## 3. Results

### 3.1. An Overview of the Pipeline

**Figure 1:**
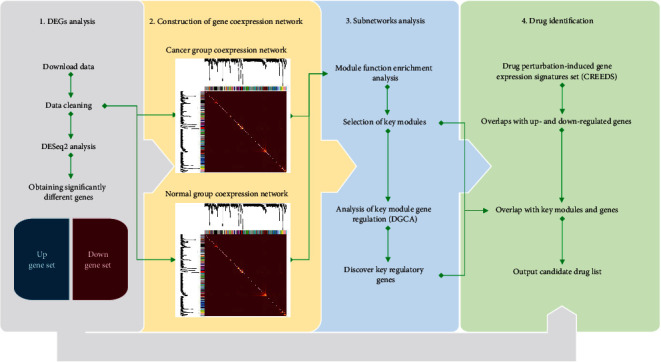
An overview of the pipeline. Four major steps are (1) differential expression genes analysis (DEGs) using TCGA dataset; (2) construction of gene coexpression network using WGCNA R packages to obtain clustering module; (3) perform functional enrichment analysis for each module, select cancer-related functional modules for subnetwork analysis, and screen for key genes; and (4) compare the gene expression characteristics induced by drug disturbances in CREEDS with DEGs, key modules, and key genes and select drugs with high overlap into the candidate list.

We take RNA sequencing data (reads counts data) from cancer and normal samples as input, and it outputs a list of candidate compounds that may help to slow aging and provide geroprotection in the corresponding tissue. It outputs a list of potential drugs, which may have potential effects and can “reverse” gene expression in cancer cells. Key genes obtained from the analysis of gene regulatory networks can be used to understand the pathogenesis of cancer and aid drug screening (preferential selection of compounds capable of regulating key genes). The detailed steps are shown in Figure 1.

### 3.2. DESeq2 Analysis Results

**Figure 2:**
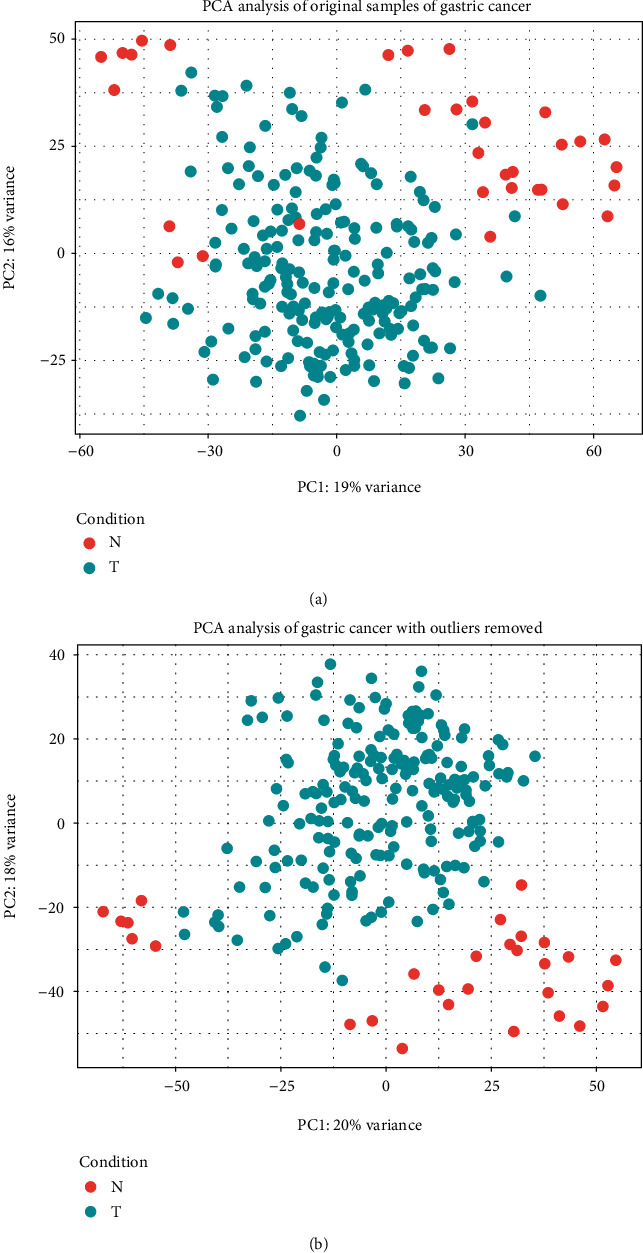
Principal component analysis (PCA) of three cancer samples. Through the matrix decomposition method, we can obtain the distribution of the samples on the principal component axis. Through the distribution, we can preprocess the sample data and remove outliers. Original sample distribution (a); filtered sample distribution (b).

The data is from the Cancer Genome Atlas (TCGA) database. It contains 242 gastric cancer samples (33 normal samples, 209 cancer samples). Principal component analysis (PCA) was performed on all samples of the three cancers to remove outliers (see Figure 2). DESeq2 is used after data processing (Supplementary Table S1). In gastric cancer samples, we obtained 9,733 significantly differentially expressed genes (5101 up-regulated genes and 4,632 down-regulated genes, compared with normal samples) (adjusted *p* value less than 0.05 was set as threshold).

**Figure 3:**
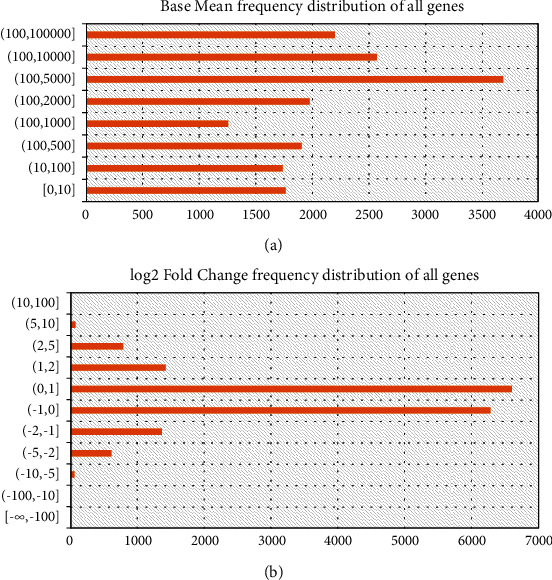
Data distribution plot for DESEQ2 results. Base mean frequency distribution of all genes (a), distribution of the mean of the gene's read counts in all samples; log2FoldChange frequency distribution of all genes (b), distribution of the mean value of the gene's fold change in all samples.

**Figure 4:**
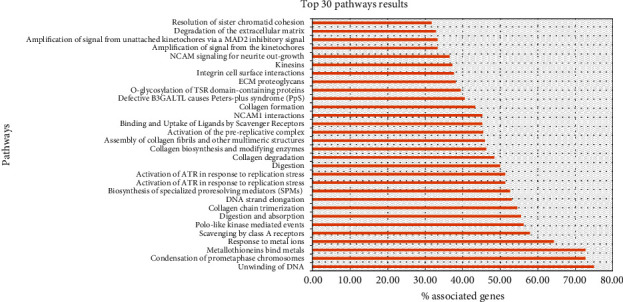
Pathway enrichment analysis results. Only the pathways with *p* value less than 0.001 are displayed. Here, we have selected the first 30 significant pathways to display.

**Figure 5:**
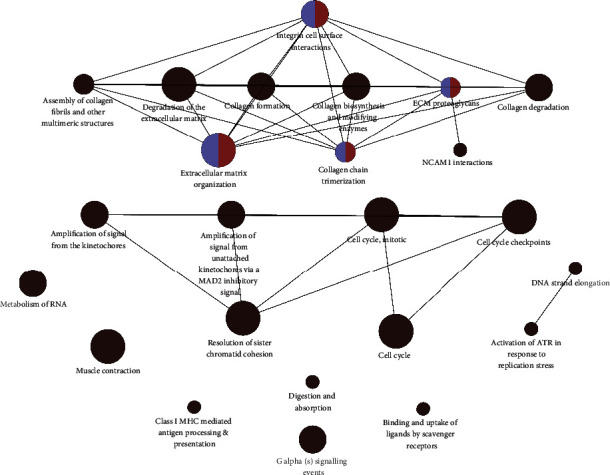
Pathways network diagram. Three sets of subnetwork diagrams are displayed.

In the case of the above threshold screening, there are many significantly differentially expressed genes. We decided to increase the screening criteria (adjusted *p* value ≤ 0.001, base mean ≥ 100, ∣ log2 fold change  | ≥1) according to the data distribution (Figure 3). We obtained 2,409 significantly differentially expressed genes (1,084 up-regulated genes and 1,325 down-regulated genes, compared with normal samples). “ClueGO” [13] software was used for pathways enrichment analysis to create and visualize networks of pathways (Figure 4). A total of 57 significantly enriched pathways were obtained (*p* value less than 0.001), and most of the pathways were mainly related to synthesis and modification (class I MHC-mediated antigen processing and presentation: R-HSA:983169, collagen biosynthesis and modifying enzymes: R-HSA:1650814, activation of the prereplicative complex: R-HSA:68962 [14], Asparagine N-linked glycosylation: R-HSA:446203 [15], etc.), cell replication (condensation of prometaphase chromosomes: R-HSA:2514853, unwinding of DNA: R-HSA:176974, DNA strand elongation: R-HSA:69190, cell cycle checkpoints: R-HSA:69620, mitotic prometaphase: R-HSA:68877, cell cycle, mitotic: R-HSA:69278, mitotic spindle checkpoint: R-HSA:69618, cell cycle checkpoints: R-HSA:69620, etc.), cell metabolism (metabolism of RNA: R-HSA:8953854, metabolism of proteins: R-HSA:392499, digestion and absorption: R-HSA:8963743, binding and uptake of ligands by scavenger receptors: R-HSA:2173782 [16], degradation of the extracellular matrix: R-HSA:1474228 [17], etc.), and signal mediation (interleukin-4 and interleukin-13 signaling: R-HSA:6785807 [18], scavenging by class A receptors: R-HSA:3000480 [19], etc.), which were highly related to the characterization of cancer. We screened some signal paths to build a network graph based on the connectivity of pathways (Figure 5).

### 3.3. WGCNA Analysis Results

**Figure 6:**
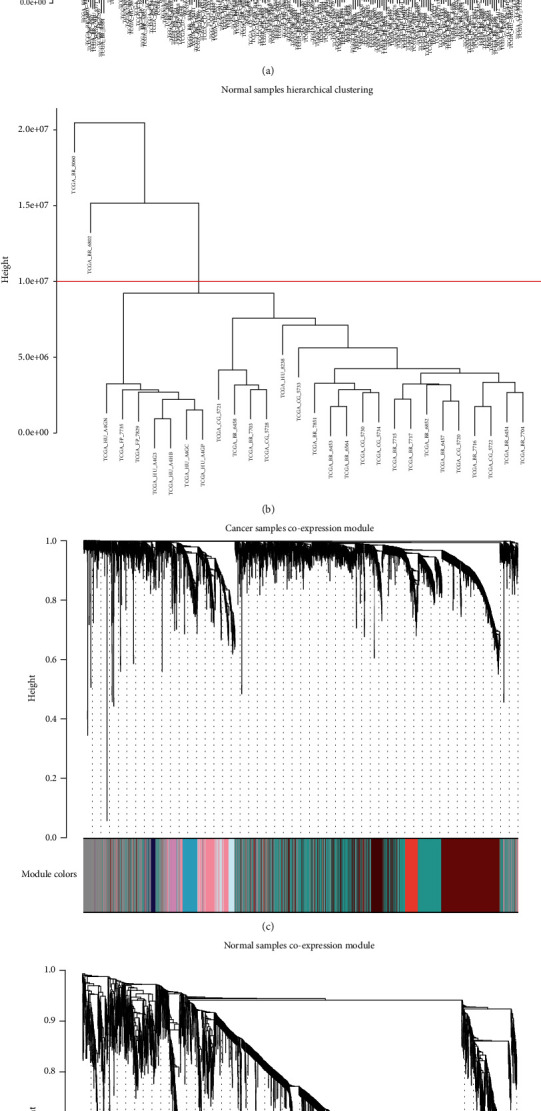
WGCNA analysis results. (a) Hierarchical clustering results of cancer samples (gastric cancer) (cut off = 0.5e^06^); (b) hierarchical clustering results of normal samples (gastric cancer) (cut off = 1e^06^); (c) cancer samples coexpression module (72 gene modules, except grey module); (d) normal samples coexpression module (36 gene modules, except grey module).

The input data of DESeq2 is also used as the input data of WGCNA. Based on the data results, we first divided the sample data into normal samples and cancer samples and run the WGCNA program separately. We remove those genes whose expression level is 0 in all samples, and then, we remove the sample points of the partial separation group based on the hierarchical clustering results of the samples (Figures 6(a) and 6(b)). There are 187 valid samples in cancer group and 27 valid samples in normal group. After running WGCNA, we obtained 72 gene modules in cancer group and 36 gene modules in normal group (Figures 6(c) and 6(d)). ClueGO cyREST tools are used for each module for functional enrichment analysis [20], which is a good batch task processing tool. The main pathway enrichment results of each module can be viewed in the Supplementary Table S2.

**Figure 7:**
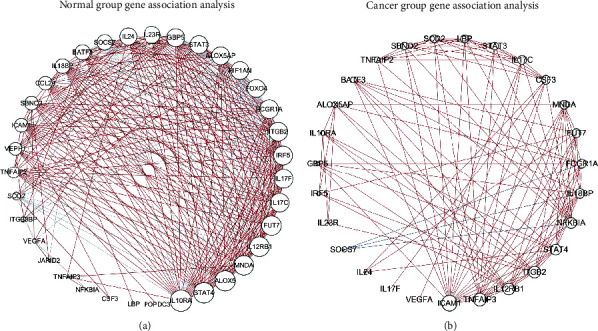
Gene association analysis by DGCA. Normal group gene association analysis (a); cancer group gene association analysis (b). Data with an absolute value of the correlation coefficient greater than 0.3 and a confidence level of less than 0.05 are shown. The node size in the figure is related to its edges, the red edges indicate a positive correlation, and the blue indicates a negative correlation.

Due to the large results, we can analyze a specific pathway, taking the interleukin-4 and interleukin-13 signaling pathway as an example. It was reported that IL-4 and IL-13 inhibited colon cancer cell-cell adhesion by down-regulation of E-cadherin and CEA molecules [20]. In the normal group, there were a total of 27 gene hits. In the cancer group, this number was reduced to 8 genes, and these genes did not overlap. The expression difference of these 35 genes in cancer and normal samples can be seen in Table 1. The correlation of these 35 genes was also calculated by DGCA: a comprehensive R package for differential gene correlation analysis [21]. We obtained the pairwise correlations of the thirty-five genes in the healthy group and the cancer group, retaining all the results of the absolute value of the correlation greater than 0.3 and the *p* value less than 0.05, and plotted the network diagram (Figure 7) through Cytoscape (https://cytoscape.org/). It is not difficult to find that related genes in the interleukin-4 and interleukin-13 signaling pathways have decreased activity in cancer samples, and their associations have also been disrupted. This may be the direction of potential drug treatment.

### 3.4. Potential Drug Discovery

**Figure 8:**
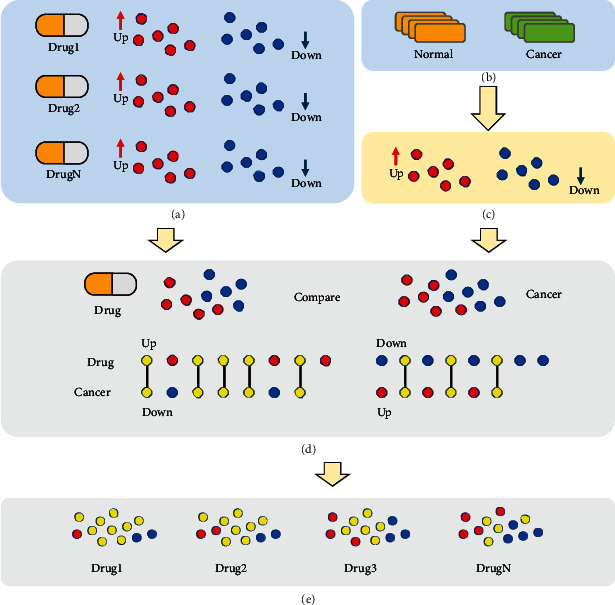
Drug screening process. (a) Expression spectrum data obtained from drug disturbance experiment; (b) cancer analysis sample data, including experiment and control group; (c) gene expression difference analysis of data in (b) to obtain gene set with significant difference expression. (d) Match the difference gene corresponding to different drugs in (a) with the gastric cancer difference gene obtained in (c) (because it is to use drugs for correction treatment, the down-regulated gene caused by drugs will match the up-regulated gene caused by gastric cancer, and the up-regulated gene caused by drugs will match the down-regulated gene caused by gastric cancer) and (e) set the corresponding threshold standard. Select the drug with high matching degree as the candidate treatment drug.

We used drug perturbation-induced gene expression signatures obtained from CREEDS to compare with genes whose gene expression was significantly (*p* value < 0.05) different in cancer and normal samples and calculated the intersection of the genes in each drug perturbation-induced gene expression signature with significantly different genes (Figure 8). The results of the comparison can be viewed in Supplementary Table S3. Because drug mining needs to be more cautious, we used stricter screening criteria (*p* value is less than 1*e* − 5) and obtained a total of 2703 matching drug perturbation-induced gene expression signatures (Supplementary Table S4). We compared the number of overlapping genes with the number of genes affected by the drug itself to obtain coverage, ranked the coverage, and selected the results with a coverage greater than 0.95 for further observation. Seven drugs were screened out, and all were found to be related to cancer treatment through verification (Table 2), and most of the genes regulated by these seven drugs were significantly differently expressed genes for gastric cancer. This may provide new ideas and directions for our use of drugs.

## 4. Discussions

In this article, we propose a new idea for cancer mechanism research and drug treatment. We use RNA sequencing data from cancer and normal samples as our input files and perturbation-induced gene expression signatures as our reference files. Through the analysis of gene expression differences, WGCNA analysis, comparison analysis of the gene disturbance characteristics affected by drugs, etc., the related pathway changes of gastric cancer were explored, and some potential drugs that highly matched the characteristics of gastric cancer gene changes were obtained. This method is very meaningful for systematically understanding the cure mechanism of gastric cancer, the changing characteristics of pathways, and medication guidance.

In the specific research process, we obtained the list of differential genes through expression differential analysis. Through certain threshold screening and GO/KEGG enrichment analysis, we obtained some potential cancer-related pathways, such as interleukin-4 and interleukin-13 signaling: R- HSA:6785807, class I MHC-mediated antigen processing and presentation: R- HSA:983169, collagen biosynthesis and modifying enzymes: R- HSA:1650814, and activation of the prereplicative complex: R- HSA:68962. In the collinearity analysis, we annotated the function of the coexpression gene network module and compared the gene association changes of the subnetwork modules including interleukin-4 and interleukin-13 signaling pathway in normal samples and gastric cancer samples. We found that interleukin-4 and interleukin-13 signaling pathways have discredited activity in cancer samples, and their associations have also been disrupted. This may be the direction of potential drug treatment.

There are a few factors that bring possible errors and uncertainties to the analyses. For example, the cancer samples collected by TCGA come from multiple individuals and multiple platforms, which will bring batch effects. In addition, gastric cancer may have multiple subtypes, which might have intrinsic differences. In the future, we will develop methods to minimize the effects of these confounding factors, and try to identify gene expression changes and pathway changes in different subtypes. As the concept of personalized medicine is proposed and promoted, the cost of next-generation sequencing is decreasing year by year. Our objective is very likely to be achieved.

## Figures and Tables

**Table 1:** Gene expression differential analysis in interleukin-4 and interleukin-13 signaling pathways.

zGroup	Gene_id	Log2FoldChange	Padj
Normal	FCGR1A	2.160463	6.85*E* − 19
	TNFAIP2	1.704628	4.34*E* − 14
	GBP5	1.813918	6.37*E* − 08
	IL18BP	0.929973	1.36*E* − 06
	FOXO4	-0.55515	1.54*E* − 05
	VEGFA	0.853914	9.28*E* − 05
	SOCS7	0.550096	0.000314
	IL17F	-1.71267	0.003176
	ITGB2	0.647619	0.010578
	STAT3	0.250713	0.018252
	IL24	0.668153	0.065269
	FUT7	-0.54276	0.076443
	IRF5	0.322352	0.103439
	IL10RA	-0.43151	0.114833
	BATF3	0.294055	0.130277
	POPDC3	-0.75591	0.130528
	VEPH1	0.581702	0.140707
	ALOX5	0.292276	0.237381
	ITGB3BP	0.152357	0.309558
	CCL24	-0.39966	0.395529
	MNDA	0.125587	0.62581
	HIF1AN	-0.03136	0.795816
	IL12RB1	-0.06538	0.826025
	ALOX5AP	-0.05154	0.866366
	IL23R	-0.06508	0.866881
	JARID2	0.016533	0.921854
	STAT4	-0.01968	0.942641

Cancer	SBNO2	0.799014	1.12*E* − 10
	LBP	3.152698	2.64*E* − 10
	ICAM1	1.209842	5.81*E* − 08
	IL17C	2.409849	1.59*E* − 06
	SOD2	0.739123	6.51*E* − 06
	NFKBIA	-0.49383	2.25*E* − 04
	TNFAIP3	0.363209	0.043201
	CSF3	0.661918	2.31*E* − 01

The results of 35 gene expression differences were extracted from the results of DESeq2. According to the WGCNA clustering results, they were divided into two groups and ranked according to the expression multiples.

**Table 2:** Evidence of drugs for gastric cancer.

Drug	Possible effects	Evidence (DOI)
Oxaliplatin	An alkylating agent that inhibits DNA replication by forming adducts between two adjacent guanines or guanine and adenine molecules	10.1007/s00280-007-0515-7
		10.1186/1756-9966-29-118
		10.1016/S0140-6736(11)61873-4

6-Alpha-methylprednisolone	Strong anti-inflammatory effect	10.1002/ddr.430020113
		10.1111/nmo.12391

Valproic acid	Inhibits tumor growth by inducing apoptosis	10.2147/DDDT.S110425
		10.3969/j.issn.1000-4718.2012.10.023
		10.1371/journal.pone.0018562

Lactam	Inhibits human gastric cancer proliferation and induces apoptosis	10.1021/ic400019r
		10.3390/molecules 18077436

Retinoic acid	Inhibit cell cycle progression	10.1046/j.1432-0436.2001.068001013.x
		10.1242/jcs.01474
		10.1111/j.1440-1746.2004.03336.x
		10.1046/j.1432-0436.1997.6150313.x

Dexamethasone	Dexamethasone not only suppressed the apoptosis-associated up-regulation of Bcl-xS but also enhanced the basal level of Bcl-xL in the cells; significantly reduces the affinity of tumor necrosis factor for gastric cancer cells	10.1016/S0014-5793(97)01083-1
		CNKI:SUN:YYDB.0.2005-07-018
		10.1007/s00464-014-3463-4

Curcumin	Inhibits proliferation of several cancer cell lines	10.4161/cbt.8.14.8720
		10.1016/j.phymed.2012.12.007
		10.3892/or.2011.1410
		10.1155/2012/915380

The drugs obtained are rigorously screened and their mechanisms discovered through the relevant literature.

## Data Availability

All the data used in this study could be downloaded from TCGA Gastric cancer transcriptome data https://portal.gdc.cancer.gov/projects/TCGA-STAD. Here, we downloaded it using R package of “RTCGAToolbox.”

## Supplementary Materials

Supplementary 1 Supplementary Table 1: results file for expression difference analysis using DESeq2 software.

Supplementary 2 Supplementary Table 2: the main pathways enrichment results of each module (cancer and normal group).

Supplementary 3 Supplementary Table 3: drug perturbation-induced gene expression signatures and expression differential gene intersection information.

Supplementary 4 Supplementary Table 4: drug perturbation-induced gene expression signatures and expression differential gene intersection information with *p* value < 1*e* − 5.

## References

[1] Bray F., Ferlay J., Soerjomataram I., Siegel R. L., Torre L. A., Jemal A. (2018). Global cancer statistics 2018: GLOBOCAN estimates of incidence and mortality worldwide for 36 cancers in 185 countries. *CA: a Cancer Journal for Clinicians*.

[2] Kumar S., Lombard D. B. (2016). Finding Ponce de Leon’s pill: challenges in screening for anti-aging molecules. *F1000Research*.

[3] Iorio F., Knijnenburg T. A., Vis D. J. (2016). A landscape of pharmacogenomic interactions in cancer. *Cell*.

[4] Ding Z., Zu S., Gu J. (2016). Evaluating the molecule-based prediction of clinical drug responses in cancer. *Bioinformatics*.

[5] Prada-Gracia D., Huerta-Yépez S., Moreno-Vargas L. M. (2016). Application of computational methods for anticancer drug discovery, design, and optimization. *Boletín Médico Del Hospital Infantil de México (English Edition)*.

[6] Lamb J. (2007). The Connectivity Map: a new tool for biomedical research. *Nature Reviews Cancer*.

[7] Dudley J. T., Sirota M., Shenoy M. (2011). Computational repositioning of the anticonvulsant topiramate for inflammatory bowel disease. *Science translational medicine*.

[8] Mirza N., Sills G. J., Pirmohamed M., Marson A. G. (2017). Identifying new antiepileptic drugs through genomics-based drug repurposing. *Human Molecular Genetics*.

[9] Duan Q., Reid S. P., Clark N. R. (2016). L1000CDS^2^: LINCS L1000 characteristic direction signatures search engine. *NPJ Systems Biology and Applications*.

[10] Wang Z., Monteiro C. D., Jagodnik K. M. (2016). Extraction and analysis of signatures from the Gene Expression Omnibus by the crowd. *Nature Communications*.

[11] Love M. I., Huber W., Anders S. (2014). Moderated estimation of fold change and dispersion for RNA-seq data with DESeq2. *Genome Biology*.

[12] Langfelder P., Horvath S. (2008). WGCNA: an R package for weighted correlation network analysis. *BMC Bioinformatics*.

[13] Bindea G., Mlecnik B., Hackl H. (2009). ClueGO: a Cytoscape plug-in to decipher functionally grouped gene ontology and pathway annotation networks. *Bioinformatics*.

[14] Lau E., Tsuji T., Guo L., Lu S. H., Jiang W. (2007). The role of pre-replicative complex (pre-RC) components in oncogenesis. *The FASEB Journal*.

[15] Contessa J. N., Bhojani M. S., Freeze H. H., Rehemtulla A., Lawrence T. S. (2008). Inhibition of N-linked glycosylation disrupts receptor tyrosine kinase signaling in tumor cells. *Cancer Research*.

[16] Yu X., Guo C., Fisher P. B., Subjeck J. R., Wang X. Y. (2015). Scavenger receptors: emerging roles in cancer biology and immunology. *Advances in Cancer Research*.

[17] Koivunen E., Ristimäki A., Itkonen O., Osman S., Vuento M., Stenman U. H. (1991). Tumor-associated trypsin participates in cancer cell-mediated degradation of extracellular matrix. *Cancer Research*.

[18] Kanai T., Watanabe M., Hayashi A. (2000). Regulatory effect of interleukin-4 and interleukin-13 on colon cancer cell adhesion. *British Journal of Cancer*.

[19] Bak S. P., Walters J. J., Takeya M., Conejo-Garcia J. R., Berwin B. L. (2007). Scavenger receptor-A–targeted leukocyte depletion inhibits peritoneal ovarian tumor progression. *Cancer Research*.

[20] Mlecnik B., Galon J., Bindea G. (2019). Automated exploration of gene ontology term and pathway networks with ClueGO-REST. *Bioinformatics*.

[21] McKenzie A. T., Katsyv I., Song W. M., Wang M., Zhang B. (2016). DGCA: a comprehensive R package for differential gene correlation analysis. *BMC Systems Biology*.

